# Transaortic removal of a large primary sarcoma from the left ventricle assisted by strategic partial resection and endoscopic guidance: a case report

**DOI:** 10.1186/s13019-024-02489-1

**Published:** 2024-01-31

**Authors:** Jae-Sung Choi, Jeongwon Kim, Se Jin Oh, You Jung Ok, Yong Won Seong, Hyeon Jong Moon

**Affiliations:** 1 Department of Cardiovascular and Thoracic Surgery, SMG-SNU Boramae Medical Center, Seoul National University College of Medicine, 20 Boramae-ro 5-gil, Dongjak-gu, Seoul, 07061 Republic of Korea; 2 Department of Cardiovascular and Thoracic Surgery, Seoul National University Hospital, Seoul, Republic of Korea

**Keywords:** Heart neoplasms, Sarcoma, Endoscopic surgery, Cytoreduction Surgical procedures

## Abstract

**Background:**

Surgical resection remains the mainstay of treatment for cardiac sarcoma, a rare but lethal disease. Achieving complete removal of a large-sized left ventricular sarcoma remains a challenge even with various surgical approaches that have been employed.

**Case presentation:**

We present a case of a 74-year-old woman with shortness of breath who underwent surgical removal of a primary cardiac sarcoma, measuring 6 × 3.5 × 3 cm, attached to the septum of the left ventricle and caused sub-aortic valve obstruction. Transaortic approach was chosen and the access to this entire huge mass was enabled by using interim partial resection which created a space for further dissection and subsequent deeper endoscopic views. The further dissection was finally able to be advanced on the apex, and the residual mass was completely resected with gross tumor-free margins.

**Conclusion:**

Interim partial resection and endoscopic guidance can highly facilitate the transaortic removal of even large left ventricular sarcomas.

**Supplementary Information:**

The online version contains supplementary material available at 10.1186/s13019-024-02489-1.

## Introduction

Cardiac malignancies can be difficult to remove due to their poor margins and infiltration into adjacent structures. Furthermore, access is very challenging, especially when the tumor is located in the left ventricle (LV) and is large in size. The lack of established standards for surgical procedures for these cases further complicates management. Left ventriculotomy is thought to be the most straightforward approache but, with a large incision, carries considerable risks of bleeding, myocardial dysfunction, or fatal ventricular arrhythmia. Transaortic, transmitral, and combined approaches have been also employed, but those cases were limited to the relatively smaller-sized tumors located in the more easily accessible sides of the LV [1, 2]. However, those approaches per se could not provide an adequate surgical view for tumors that extended toward the apical and/or mitral subvalvular region. In this report, we present a rare but representative case of complete transaortic removal of a large LV sarcoma using interim partial resection and endoscopic guidance.

## Case presentation

**Fig. 1:**
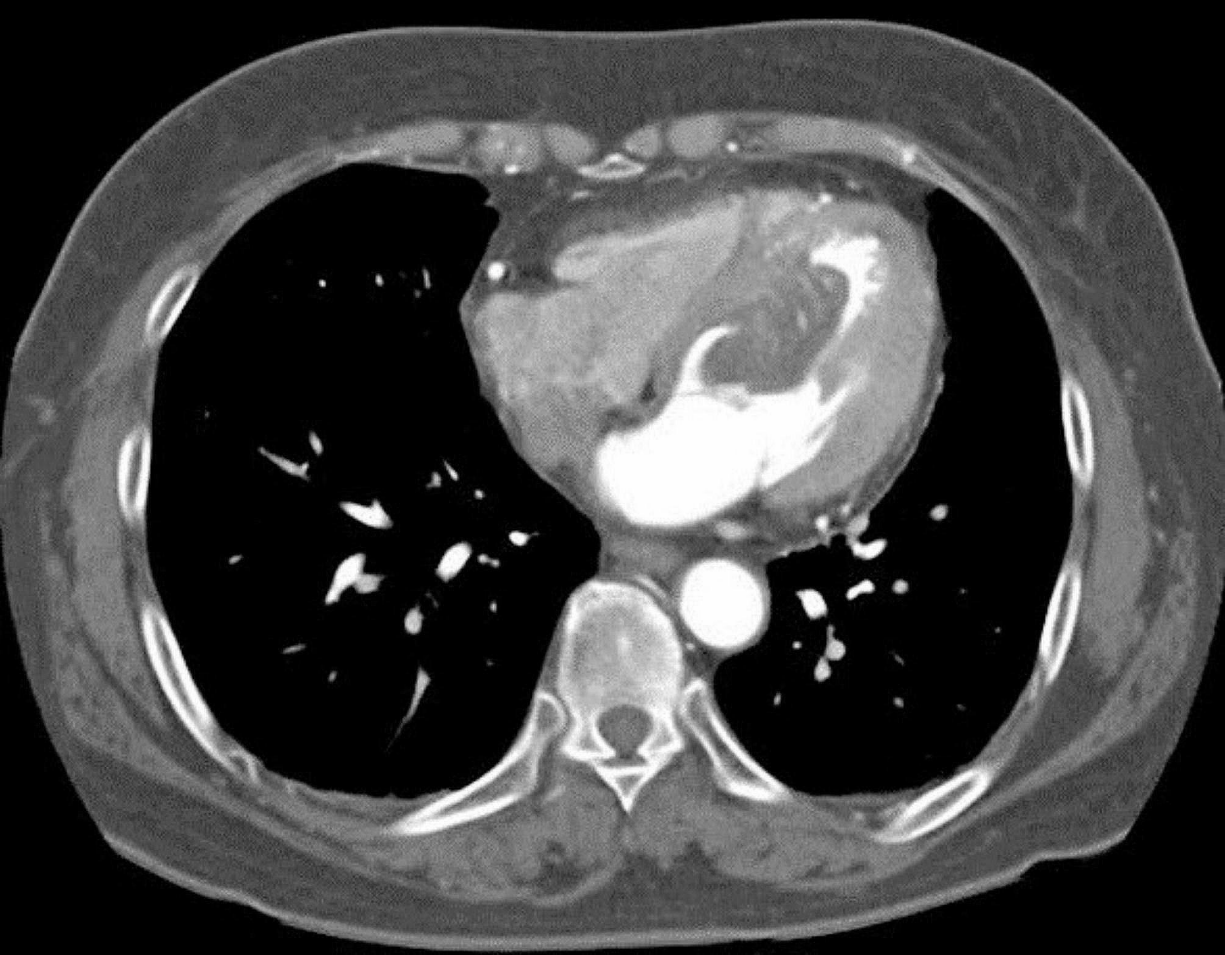
Initial coronary computed tomography angiography showing a mass adherent to the septum in the left ventricular cavity

A 74-year-old woman with a history of essential tremor and dyslipidemia presented to the emergency department with shortness of breath. Her electrocardiogram and laboratory panel were normal, but coronary computed tomography (CT) angiography showed a large low attenuation mass on the LV septum (Fig. 1) though there was no significant stenosis in coronary arteries. Transthoracic echocardiography (TTE) revealed severe subvalvular stenosis of the aortic valve (AV) but preserved other valves and LV function. In cine magnetic resonance imaging, the mass was found to be attached to the LV septum, causing dynamic LV outflow tract obstruction (Supplementary video 1).

**Fig. 2:**
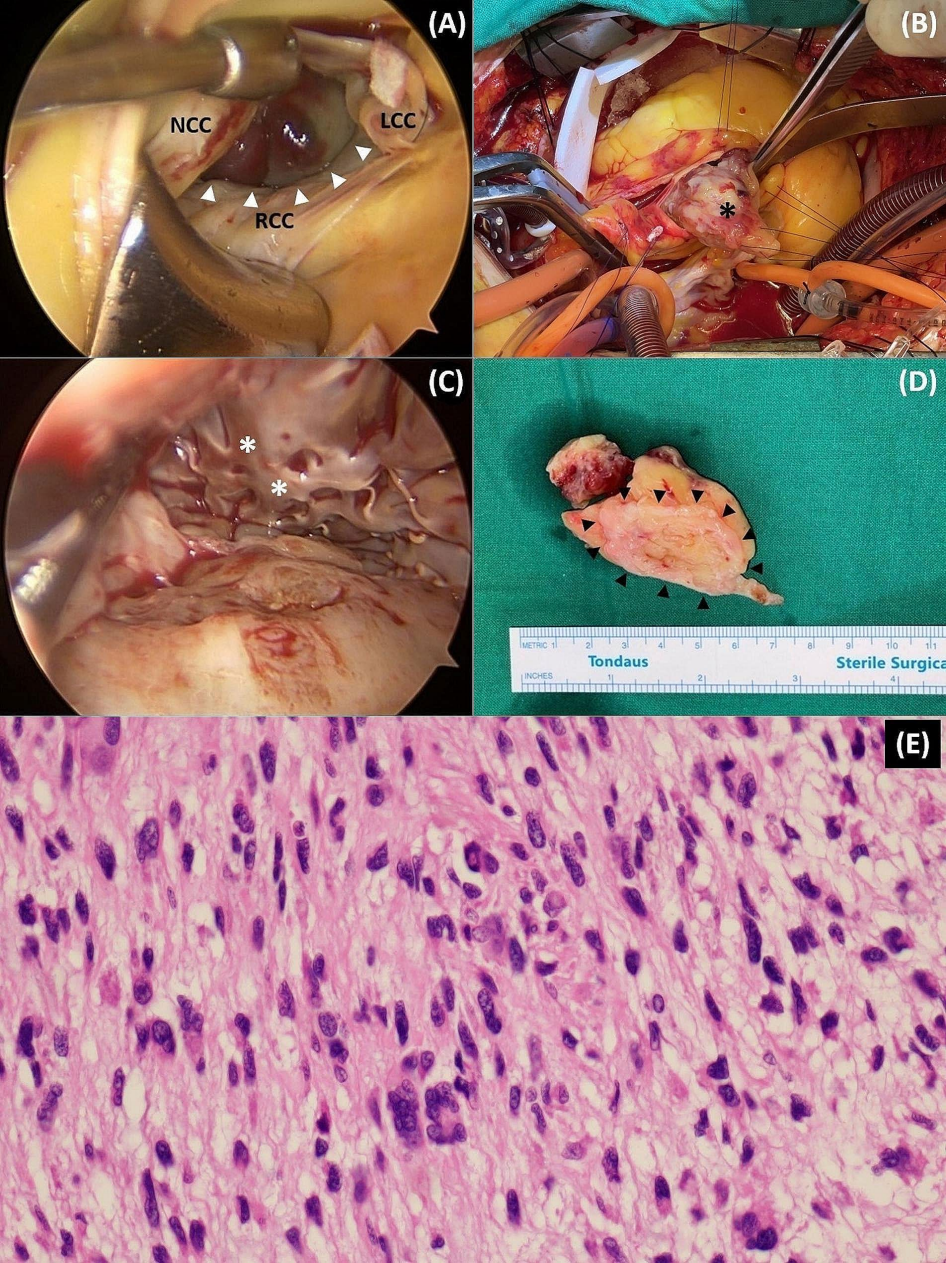
Intraoperative thoracoscopic view and gross appearance of the mass. **(A)** Upper pole of the mass in dark red color (white arrowheads) seen through retracted aortic leaflets. **(B)** Huge residual LV mass (black asterisk) being removed through the aortic valve. **(C)** Thoracoscopy shows the apex of the left ventricle (white asterisks) and a clear site of the interventricular septum (white arrows) after resection of the mass. **(D)** Well-demarcated attachment site of the mass (black arrowheads). **(E)** Pleomorphic cells with abundant cytoplasm, mitotic features of high-grade sarcoma (H&E, x400)

An urgent operation was performed the following day using a median sternotomy and cardiopulmonary bypass with bicaval cannulation in case we need a combined approach through both the aortic and mitral valves. After the aortic cross-clamping, a “hockey-stick” aortotomy was following the infusion of antegrade del Nido cardioplegic solution. The aortic valve was intact but, the LV outflow tract was obstructed by the LV mass. A partial oblique resection of the mass was first performed to help create a space for further dissection and to obtain deeper endoscopic views. This strategic interim partial resection also facilitated the removal of the entire mass, which would have otherwise been challenging to extract through the relatively much smaller AV orifice. The lowest rectal temperature was 31 °C. The CPB and ACC times were 141 and 76 min, respectively. A 5-mm thoracoscope was used to aid in visualizing the tumor margin. The attached portion of the residual tumor could be safely and clearly dissected out from the LV septum using Metzenbaum scissors and a suction tip (Fig. 2A-C). On gross examination, the tumor size was 6.0 × 3.5 × 3.0 cm, and it had an irregular and smooth surface with a rubbery to firm consistency (Fig. 2D). Frozen section biopsy indicated increased cellularity and spindle cell proliferation, suggesting a malignant rather than benign tumor. The entire mass was confirmed to be a cardiac sarcoma upon pathological examination (Fig. 2E).

The patient’s postoperative course was uneventful. She stayed in intensive care unit for one day and was discharged on postoperative day 7. Follow-up chest-CT, fluorodeoxyglucose-positron emission tomography, and TTE were performed a month after discharge and showed no abnormal hypermetabolic lesion suggesting metastasis or residual intracardiac malignancy. However, the patient rejected the recommended adjuvant radiation therapy. Five months later, a follow-up chest-CT revealed the development of an 11 mm-sized nodule in the LV septal wall (Supplementary Fig. 1), but the patient reported no related symptoms and still refused further treatment.

## Discussion

The authors successfully removed a large LV sarcoma through the AV orifice using interim partial resection and endoscopic guidance. The use of endoscopic guidance provided a clearer and more accurate surgical view, facilitating the complete removal of the mass with minimal damage to adjacent normal heart structures. The interim partial resection technique also provided more space for manipulation of the thoracoscope, sucker handle, and dissecting scissors, eliminating the need for additional procedures such as left ventriculotomy or mitral valve exposure.

Complete surgical resection is the main factor associated with higher survival rates in primary cardiac sarcomas, making precise and thorough resection essential [3]. In order to achieve this goal, the application of endoscopic guidance appears to be useful. A systematic review of the utilization of cardiac endoscopy, which included 34 studies incorporating 54 subjects with LV tumors, thrombus, or myocardial hypertrophy, showed that complete resection of LV lesions could be achieved in all cases [4]. Although there is no established standard surgical procedure for the removal of LV masses, previously reported approaches include (1) transmitral valve through the left atrium, right atrium, and atrial septum, or autotransplantation in cases of malignant tumor [5], (2) transaortic valve through the ascending aorta with or without video-assistance [6, 7] and (3) through a small longitudinal incision in the LV [8]. However, the majority of the successful approaches were for benign tumors such as myxoma. When it comes to sarcomas, which attach to the large endocardial surface and are unfavorably located, such as at the ventricular septum, as in our case, complete and safe resection can be challenging with those conventional techniques.

This report showed that interim partial resection and endoscopic guidance provided easier access to the attachment site and increased the chance of complete and controlled removal of even a large sarcoma only through the aortic valve. This approach reduces concerns about injury to the mitral subvalvular apparatus, postoperative myocardial dysfunction, or ventricular arrhythmia. While no comments have been made about the comparison between rigid and flexible endoscopes, interference between the endoscope and other instruments such as forceps and scissors, regardless of the type of endoscope [9], can be an issue if there is very limited free space left for the instruments due to the large space-occupying tumor, which tends to be the case with malignancy rather than benign tumors. In this situation, a well-designed strategic partial resection can address this problem nicely. This partial resection, followed by saline irrigation, can be safely carried out for solid tumors such as sarcomas.

## Conclusion

The present report highlights the feasibility and safety of interim partial resection and endoscopic guidance for transaortic complete removal of even large LV sarcomas.

### Electronic supplementary material

Below is the link to the electronic supplementary material.

Supplementary Material 1. Supplementary video 1. Initial cardiac cine magnetic resonance imaging showing back-and-forth movement of the obstructing mass through the aortic valve, which is adherent to the septum of the left ventricle.

Supplementary Material 2. Supplementary Fig. 1 A follow-up CT scan image taken 5 months after the operation revealing the interval development of an intraluminal, low attenuating nodule protruding from the left ventricular septal wall.

## Abbreviations

AV: aortic valve
CT: computed tomography
LV: left ventricle
TTE: transthoracic echocardiography

## References

[1] Keeling I, Oberwasser P, Rigler B (1999). Transaortic access for excision of a left ventricular myxoma. Ann Thorac Surg.

[2] Kawano H, Tayama K, Akasu K, Komesu I, Fukunaga S, Aoyagi S (2000). Left ventricular myxoma: report of a case. Surg Today.

[3] Isambert N, Ray-Coquard I, Italiano A, Rios M, Kerbrat P, Gauthier M (2014). Primary cardiac sarcomas: a retrospective study of the French sarcoma group. Eur J Cancer.

[4] Soylu E, Kidher E, Ashrafian H, Stavridis G, Harling L, Athanasiou T (2017). A systematic review of left ventricular cardio-endoscopic surgery. J Cardiothorac Surg.

[5] Bruckner BA, Saharia A, Aburto J, Reardon MJ (2009). Delayed left ventricular free-wall rupture after cardiac sarcoma resection. Tex Heart Inst J.

[6] Walkes JC, Bavare C, Blackmon S, Reardon MJ (2007). Transaortic resection of an apical left ventricular fibroelastoma facilitated by a thoracoscope. J Thorac Cardiovasc Surg.

[7] Tsukube T, Okada M, Ootaki Y, Tsuji Y, Yamashita C (1999). Transaortic video-assisted removal of a left ventricular thrombus. Ann Thorac Surg.

[8] Abad C, Novoa J, Delgado A, Alonso A (2014). Myxoma of the left ventricle. Tex Heart Inst J.

[9] Reuthebuch O, Roth M, Skwara W, Klövekorn W-P, Bauer EP (1999). Cardioscopy: potential applications and benefit in cardiac surgery. Eur J Cardiothorac Surg.

