# Supplementary figures and images for: Transaortic removal of a large primary sarcoma from the left ventricle assisted by strategic partial resection and endoscopic guidance: a case report
Source: J Cardiothorac Surg. 2024 Jan 31;19:34. doi: 10.1186/s13019-024-02489-1 (PMC10829275; doi:10.1186/s13019-024-02489-1)

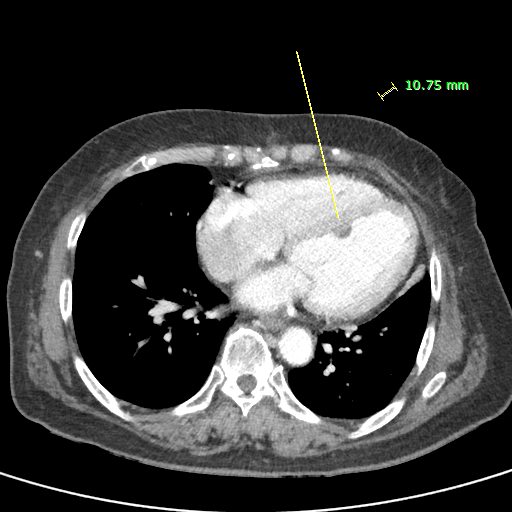

Supplement: Supplementary file 2 — Supplementary Material 2. Supplementary Fig. 1 A follow-up CT scan image taken 5 months after the operation revealing the interval development of an intraluminal, low attenuating nodule protruding from the left ventricular septal wall. [file 13019_2024_2489_MOESM2_ESM.tif]
